# Applying a framework for assessing the health system challenges to scaling up mHealth in South Africa

**DOI:** 10.1186/1472-6947-12-123

**Published:** 2012-11-05

**Authors:** Natalie Leon, Helen Schneider, Emmanuelle Daviaud

**Affiliations:** 1 Health Systems Research Unit, Medical Research Council of South Africa, P.O Box 19070, Tygerberg, Cape Town, 7505, South Africa; 2 School of Public Health, University of the Western Cape, Private Bag X17, Bellville, Cape Town, 7535, South Africa

**Keywords:** MHealth, Mobile phone technology, Health systems framework, Community-based health services, South Africa

## Abstract

**Background:**

Mobile phone technology has demonstrated the potential to improve health service delivery, but there is little guidance to inform decisions about acquiring and implementing mHealth technology at scale in health systems. Using the case of community-based health services (CBS) in South Africa, we apply a framework to appraise the opportunities and challenges to effective implementation of mHealth at scale in health systems.

**Methods:**

A qualitative study reviewed the benefits and challenges of mHealth in community-based services in South Africa, through a combination of key informant interviews, site visits to local projects and document reviews. Using a framework adapted from three approaches to reviewing sustainable information and communication technology (ICT), the lessons from local experience and elsewhere formed the basis of a wider consideration of scale up challenges in South Africa.

**Results:**

Four key system dimensions were identified and assessed: government stewardship and the organisational, technological and financial systems. In South Africa, the opportunities for successful implementation of mHealth include the high prevalence of mobile phones, a supportive policy environment for eHealth, successful use of mHealth for CBS in a number of projects and a well-developed ICT industry. However there are weaknesses in other key health systems areas such as organisational culture and capacity for using health information for management, and the poor availability and use of ICT in primary health care. The technological challenges include the complexity of ensuring interoperability and integration of information systems and securing privacy of information. Finally, there are the challenges of sustainable financing required for large scale use of mobile phone technology in resource limited settings.

**Conclusion:**

Against a background of a health system with a weak ICT environment and limited implementation capacity, it remains uncertain that the potential benefits of mHealth for CBS would be retained with immediate large-scale implementation. Applying a health systems framework facilitated a systematic appraisal of potential challenges to scaling up mHealth for CBS in South Africa and may be useful for policy and practice decision-making in other low- and middle-income settings.

## Background

There is widespread enthusiasm for the use of mobile phone technology to improve health services (or mHealth), but evidence is limited on how to implement mHealth effectively at scale in health systems. This paper applies a health systems perspective to guide analysis of potential challenges of scaling up mHealth for the monitoring and evaluation (M&E) of community-based health services (CBS) in South Africa.

The growing enthusiasm for mHealth is driven not only by the demonstrated benefits of mobile technology, but also by the widespread availability of mobile phones, and the relatively low levels of literacy required to use them [1-4]. South Africa is amongst countries with the highest proportion of mobile phone users per population, with 93 out of 100 people being subscribed to a mobile phone network [5-8]. There is also considerable interest in the broader use of mobile phone technology within government, with the health department recently developing an eHealth strategy inclusive of mHealth [9]. In a parallel initiative the South African primary health care system (PHC) is undergoing a process of revitalization in which the currently fragmented community-based ‘outreach’ functions are being prioritised for greater development, through integration with and management by the health department [10]. This formalisation of CBS is expected to bring greater standardisation of M&E and supervision systems for community health workers (CHWs). The National Department of Health (NDOH) wanted guidance about how mobile phone technology could support the newly integrated, nation-wide community-based health service, which prompted this investigation.

Benefits of mobile phone technology derive from the rapid collection, transmission, storage and transformation of data which allow for timely access to data, real time monitoring of data gathering and programme activity, rapid analysis and auto-generated reporting. Mobile phone technology also offers the benefit of communication with individuals and large groups, through for instance, instant messaging. These functions have been put to use in various applications of mHealth such as research and disease surveillance, to support M&E, supervision, planning and development of service delivery, as tools for decision-making in clinical services, in health promotion and disease prevention and in education of health professionals (see Table 1).

**Table 1:** Different applications of mHealth in community-based health care settings

**mHealth applications**	**Examples**
Data collection	· Electronic tools for data collection and rapid access to data for purposes of research and disease surveillance.
Management	· Management of health information for planning, monitoring, evaluation and supervision of workers and service delivery.
	· Administrative help with data collection and rapid reporting.
	· Facilitating communication amongst community health workers (CHWs) and between CHWs and supervisors.
	· Improving administrative systems, for example, for human resource, financial and supply chain management.
Clinical service delivery	· Providing support for health workers at point of care for diagnosis and treatment, via job aids and decision-making tools in the form of electronic guidelines, algorithms and referral mechanisms.
	· Patient electronic health records that can be accessed by both community and facility-based health personnel.
	· Patient access to medication via electronic prescribing system.
Health promotion activities	· Health promotion messaging via mobile phones directly to patients to increase health awareness, support treatment adherence or promote access to health services.
	· Audiovisual applications available on mobile phones to use as a job aid for CHWs.
	· Support for scheduling of home visits and targeted advice.
Education and training	· Training personnel via distance learning opportunities.
	· Evaluation of the impact of the education through distance quizzes.
	· Ongoing training through regular electronic updates and access to reference material.

Despite the widespread enthusiasm for and use of mHealth, a number of reviews in recent years have drawn attention to gaps in evidence on the impact of mHealth at scale, the main limitation being the small scale of projects [2-4,6,11]. Also, not enough is known about the social, organisational and cultural elements of successful implementation and adoption of information and communication technology (ICT) [12,13], a gap in knowledge that extends to mHealth.

Most mHealth interventions considered successful in low- and middle-income countries (LMICs) are based in non-governmental organisations (NGOs) and not integrated into the mainstream of public health services [4]. The challenges of system integration are compounded by the fact that evaluations of mHealth (and eHealth) interventions tend to focus on feasibility, rather than impact and cost-effectiveness, making it difficult to conclude on benefits [4,13-15]. Projects using mobile phone technology for data collection and monitoring have compared data quality, accuracy, time, training required and costs between traditional paper and pen methods and the new mobile technology. The focus is on intermediary outcomes, such as effects on convenience and efficiency of information management and does not extend to how this might be impacting on quality and efficiency in terms of improving service delivery processes, strengthening health systems and improving health outcomes [3,4].

Reviews of mHealth projects in LMICs have identified a range of challenges with implementation which would have implications for the up-scaling of such projects. Many of the barriers go beyond the complexity of the mobile technology itself and are related to broader health systems challenges – in the practices of health personnel, the integration of new technology with existing information systems, sustainable funding and appropriate leadership to steer these shifts [2-4,6,11,14-21]. Using the case of community-based health services in South Africa, we developed and applied a health systems framework to appraise the potential opportunities and challenges to effective implementation of mHealth at scale in health systems.

## Methods

The methods included interviews with key actors in the field of mHealth, assessment of three local mHealth projects and a review of grey and indexed literature. Interviews were conducted with nineteen key informants from organisations involved in mHealth programmes in South Africa. They included research organisations, NGOs and providers of mobile phone technology systems (also referred to as digital providers). Table 2 shows the number and types of organisations that participated in the research and the number of key informants interviewed. Organisations were selected based on their involvement with mobile phone technology for M&E of community-based services and/or involvement with the provision of mobile phone technology services. The initial list of individuals and organisations was expanded through snow ball sampling until a point of saturation was achieved. Key informants knowledgeable of their organisation’s use of mobile technology were selected for interviews.

**Table 2:** Description of types and numbers of participating organisations and key informants (KIs)

**Types of organisations**	**No. organisations (No. KIs)**
Research organisations using mobile phones for community-based research projects and or evaluating its use in community-based health care settings.	3 (6)
Non-governmental organisations (NGOs) using (or planning for the use) of mobile phones for the delivery and/or monitoring and evaluation of services.	4 (7)
Developers and providers of mobile phone management systems (for profit and non-profit organisations).	4 (5)
Other: An NGO providing management support for community–based NGOs.	1 (1)
Total	12 (19)

Three local mHealth projects were purposefully selected following the key informant interviews to assess how different software applications were used in research and service delivery settings. The review of projects drew on interviews with management and with digital providers, site visits where the mobile technology management systems were demonstrated and document reviews. Finally, indexed and grey literature on the use of mHealth in CBS was reviewed to understand the context, scope, purpose and effectiveness of the mobile phone technology used in various projects in South Africa and other low- and middle-income country settings.

Interviews were conducted between August and November 2011, using a semi-structured interview schedule that explored the organisations’ use of mobile phone technology, the implementation processes and the benefits and challenges. Interviews with the digital providers explored their experiences with the development and implementation of the software and data management systems.

The three case examples of mHealth applications discussed in more depth include the use of mHealth for:

Data collection and management of a community-based intervention research study conducted by the Health Systems Research Unit (HSRU) of the Medical Research Council of South Africa (MRC).

Real-time monitoring the activities of lay health workers spread across facilities from a central point, by a social media NGO that provides country-wide health education and awareness services in clinic waiting rooms and in community-based settings.

Routine data collection for M&E of community-based health services by two community health care organisations.

Interviews were digitally recorded and recordings were used to supplement notes taken during the interviews. Case examples were described according to key features that included the setting, purpose and nature of the mHealth solution, software development, funding sources and whether the mHealth intervention was evaluated. The elements, benefits and challenges were compared and contrasted. These were then categorised and discussed under the four main areas of the health systems framework described below.

Taking the local experiences and broader challenges identified in the literature as starting points, a framework was developed to appraise the health systems challenges of implementing mHealth for CBS at scale. The proposed framework includes an assessment of the stewardship, organizational, technological and financial dimensions of systems. It formed the basis for a theoretical analysis of the system challenges South Africa may face in up-scaling mHealth for CBS. Through the use of the conceptual framework and the challenges identified by the case examples, interviews and the literature elsewhere, we interpreted what we understood to be the challenges of implementing mHealth at scale. From this the main recommendations to inform policy and practice decisions were generated.

Ethical approval for the study was granted by the Ethics Committee of the University of Western Cape. Key informants were required to give written informed consent.

## Results and discussion

### Developing a health systems framework for decision-making about mHealth for CBS

**Figure 1:**
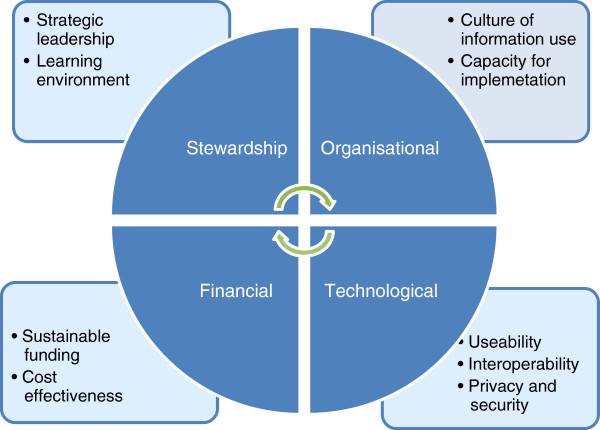
**Health systems framework for decision-making about mHealth for community-based health services.** The framework adapts and draws on three existing approaches to reviewing various eHealth applications. Figure 1 illustrates the four health systems dimensions that should be considered when applying a health systems perspective to appraise the challenges of scaling up mHealth; stewardship and organisational, technological and financial systems. Each dimension has two or more elements that are important to consider when making decisions about acquiring and or scaling up mHealth.

Against the background of multiple systems challenges identified in the literature, we identified the need for a framework with a more explicit focus on the health system dimensions of implementing mHealth. We developed a health systems framework to guide our reflection on the potential challenges of scaling up mHealth for the monitoring and evaluation of CBS in the South African setting. The framework, illustrated in Figure 1 and detailed in Table 3, has four main, interconnected health system dimensions, each with two or more key elements that should be addressed when making decisions about mHealth implementation. These are government stewardship, organisational systems, technological systems and financial systems.

**Table 3:** A Health systems framework: Health system dimensions required for scaling up mHealth for community based health services

**Health system dimension**	**Health system capacity requirements**
**Government stewardship: Is there a policy environment supportive of mHealth?**	**· Strategic leadership:** Strategic leadership is present through policy guidelines that promote alignment with strategic health goals, funding sources, common ICT standards and partnerships for collaboration nationally and internationally.
	**· Learning environment:** Government stewardship includes creating a learning environment, where projects are evaluated systematically and where collaboration and sharing of knowledge can contribute to a central repository of evidence on mHealth, which in turn can influence policy and practice.
**Organisational: Is there a culture of and capacity for using information technology for management?**	**· Capacity for implementation:** The health system has the capacity, managerially and technologically, to successfully implement mHealth interventions. This includes assessment of eReadiness, a functional ICT environment and effective mechanisms for implementation, support and monitoring and evaluation.
	**· Culture of information use:** There is an organizational culture and tradition of using health information for management - to ensure that the increased access to electronic information is used for quality improvements in health services.
**Technological: How useable, integrated and sustainable is the chosen technology?**	**· Use-ability:** The technology has ease of use, flexibility and durability and end users experience the new technology as benefiting their work.
	**· Interoperability:** Interoperability of information systems ensures there is smooth communication across technological and information platforms as well as smooth integration with existing work practices. Common standards (required for interoperable systems) are developed through consensus amongst the multiple stakeholders including health ministries, digital providers, health management, clinical staff, patients, and funders. The relative merits of open source versus proprietary software are addressed as this has implications not only for interoperability, but also for financial sustainability.
	**· Privacy and security:** Privacy and security of data is ensured. Additional regulations for protecting electronic data may be required to secure privacy of data.
**Financial: Is adequate financial provision being made for the medium to long term use of mHealth?**	**· Sustainable funding:** Securing sustainable funding for large-scale implementation is a major requirement and requires clear business and funding plans. Plans should be realistic, especially as ICT projects may cost more and take longer than initially planned.
	**· Cost-effectiveness:** The cost-effectiveness of mHealth strategies is evaluated. mHealth interventions are weighed up against other priority and evidence-based interventions (in terms of the costs, resources and capacity requirements), and opportunity costs are routinely considered. Unintended consequences of introducing new technology within a weak health system are monitored to minimize negative effects.

The proposed health systems framework adapts and draws on three existing approaches to reviewing implementation of various eHealth applications. Ali & Bailur [19] in their analysis of the prerequisites for sustainable ICT development in LMICs, identified five categories, namely institutional/organisational, social/cultural, financial, technological, and environmental sustainability, with key questions to be addressed in each category.

Bukatchi and Pakenham-Walsh [11], in their review of ICT in LMICs, highlighted the importance of infrastructural and cultural context and systems challenges. They noted:

*“ The implementation of health ICTs in developing countries and in sub Saharan Africa in particular has been hampered by traditional obstacles: poor infrastructure; lack of resources; and insufficient political commitment and support ”.*[11]

The authors summarised this as the ‘Four Cs” where variation usually occurs: ‘Culture’ of information and technology use, ‘Capacity’ to manage effective implementation, use, and maintenance of the new information technology, ‘Connectivity’ which refers to the interlinking or interoperability of information and technology systems and the ‘Costs’ or financial implications.

Finally, an eReadiness assessment tool developed by Khoja and colleagues [20] highlight similar areas for review: readiness in planning, technology, learning, societal and policy spheres. The three approaches described above do not focus on mHealth exclusively, but rather on various other eHealth and telemedicine applications. Our framework re-configured these various categories and presents them in simple, but comprehensive health system terms. It places the emphasis more squarely on the appraisal of health system dimensions for guiding informed decisions about the acquisition and implementation of mHealth at scale. The framework does not assume that implementing mHealth is the inevitable outcome of the early decision-making process - as would be the expectation of some readiness assessment tools and staging models used, for instance, in telemedicine [20,22]. Nevertheless, decision-making and implementation processes are not static, and this framework may also be useful when mHealth programmes are already implemented, to review progress and to optimize mHealth interventions.

The dimensions of the health system framework are detailed in Table 3, with an overarching question and key elements of systems capacity spelt out. Whilst the focus in this paper is on monitoring and evaluation strategies for CBS, these health system requirements are common to other eHealth and mHealth interventions, and have been well documented in related areas in Africa, Asia and Latin America [1-4,6,11,15,18,19,21,23-25]. We have thus also drawn from the lessons and recommendations from these reviews, using this as a set of criteria for appraising the challenges for scaling up mHealth for monitoring and evaluation of CBS in South Africa (Table 3).

### Applying the health system framework to current examples of mHealth for CBS in South Africa

There have been several studies documenting the feasibility and benefits of mHealth technology in South Africa, including the use of personal digital assistants (PDAs) and mobile phones for research and for managing and monitoring community-based social and health services [8,26-30]. There have also been studies of mHealth applications for health promotion and adherence support [7,8,27,29-33]. In a more recent application, mobile phones have been used as a tool for supporting the delivery of an integrated community-based health care package of services aimed at saving newborn lives in a large randomised controlled trial [28]. Although none of these projects were implemented at scale, they point to the potential benefits of mobile phone technology for CBS in South Africa. Table 4 collates the findings from key informant interviews and document reviews and describes the setting, purpose, details and funding of each of the three mHealth applications used by three organisations in South Africa.

**Table 4:** Three case examples of mHealth applications in community-based services in South Africa

**Example Organisation**	**Community-based services research Medical Research Council of South Africa (MRC): Health Systems Research Unit**	**Monitoring of facility based lay workers NGO: Community Media Trust (CMT)**	**Information collection on community-based services NGOs: Choice Trust and Valley Trust**
**Setting**	· The Good Start research study, conducted by MRC. The study was a large scale community-based, cluster RCT promoting ante- and post natal care in rural and peri-urban areas in Western Cape and Kwazulu-Natal provinces.	· A social-media NGO providing health awareness education and treatment literacy at clinics and schools throughout South Africa.	· Two community-based health care NGOs in the Limpopo and Kwazulu-Natal provinces, providing health promotion and prevention services for HIV, TB and chronic disease.
**Purpose of mHealth application**	· Daily data collection and monitoring of community health worker (CHW) activities.	· Increase efficiency of monitoring and supervision of treatment literacy practitioners (TLPs).	· Feasibility and efficiency of a mobile management software application, for monitoring and evaluation of CBS, compared to a paper-based monitoring system.
	· Management and supervision of large numbers of CHWs.		
**Nature of mHealth application**	· Mobile phones combined with web-based interface for data collection, management and supervision.	· Mobile phones used for electronic reporting of core indicators in daily work.	· CHW electronic data collection from any location with immediate transmission to their supervisors.
	· Focus on recruitment of study mothers, monitoring the fidelity of the intervention delivery, and managing caseloads and reporting.	· TLPs filled in their pre-loaded work log sheet on their mobile phones from any location and immediately transmitted it to a web-based consol.	· CHWs used a patient specific bar code to log into the mobile management system via the phone, to update patient records during home visits.
	· CHWs issued with entry-level mobile phones with pre-loaded electronic survey forms for data capturing.		
	· Able to immediately enter data collected from their allocated households and transmit completed forms via mobile phones, using internet connectivity, to a central computer server.	· Allowed managers to access, aggregate, analyse, correct errors and report on TLPs’ activities more quickly.	· Data captured and stored on custom-designed web-based patient and workforce management system accessible to supervisors.
	· Supervisors used a custom-designed management console (or computer terminal) for daily access to data, monitoring and planning of CHW activity.	· A management console allowed for aggregation of data and auto-generated management reports.	· Allowed doctors and nurses at clinic level to interact with the data using a web browser, to monitor patient follow-up.
**Software development**	· The electronic survey form and management console developed in collaboration with a for-profit digital provider, using a proprietary software application called Mobenzi Researcher.	· A software system, called Capture, was developed by a non-profit digital provider, using open-source software.	· A software system, called Nompilo, was developed by a for-profit digital provider, using proprietary software.

The organisations in the three case examples used mobile phone technology to electronically report on core process indicators of their daily work, such as registration of households, assessment of household health needs, recording details of home visits and facility educational sessions, and reporting to supervisors and managers. A key feature of the technology was the use of mobile phones in combination with a web-based interface that provided for the aggregation of electronic data from mobile phones onto a management ‘console’ which could then be accessed by supervisor and managers and if need be, by health facility personnel. This provided for a range of tools for supervisors and managers to improve their efficiency. The organisations all reported similar benefits related to an increase in convenience and efficiency of data collection, transfer, storage and analysis and management of data, as compared with paper-based systems. In the research project for instance, supervisors could in real-time, access the completed surveys and do quality checks of the data and they could auto-generate reports - shortening the time from data collection to final reporting on results.

Other benefits reported include that the costs and delays of using paper-based data collection were removed. For the service delivery NGOs, the rapid access to data on CHW activities meant less time spent on aggregation of data, improved mechanisms for data quality checks, auto generated reports and reduction of backlogs in reporting. Managers reported that these information gathering benefits in turn allowed for increased efficiencies in monitoring and evaluation, supervision, management and planning. An added advantage was that mobile management systems allowed for two-way communication between supervisors and CHWs (e.g. updating forms and home visit schedules).

Below we applied the health systems framework to review the experiences of mHealth interventions in these organisations and to highlight the differences between these and large-scale projects required for mainstream health service delivery.

#### Stewardship

In all three instances, projects were initiated by the organisations themselves rather than by the health department. The interventions were on a small-scale and not integrated with the broader public health system and consequently did not require government level policy support. The management of these organisations provided strategic leadership and they ensured that the mHealth intervention was aligned with their goals - mainly to determine if mobile phone systems could increase efficiency of data collection and monitoring of CHWs.

#### Organisational systems

As described earlier, all three organisations reported that the rapid access to data allowed for increased efficiencies in the management of their information flow and supervision of community health workers. The major benefit was a reduction in time from data collection to researchers and managers accessing the data, shortening the time required for aggregating, analyzing and reporting. For example, in the evaluation of the Choice and Valley Trust use of mobile phones, the utilisation of the electronic solution was more advantageous when compared with the paper-based system. With electronic solutions CHWs were able to collect information in half the time required for a paper-based system [29]. Another benefit was that the management tools allowed for more efficiently monitoring of the work processes of CHWs (such as updating and checking progress with home visitation schedules). The research manager described the substantial reduction in time from data collection to data analysis and reporting and commented that she “could not imagine doing future community-based research projects without using mobile phones”.

The organisations reported few implementation problems. Training of CHWs, the front end users of the phones, took less than a day with occasional updates as most CHWs are familiar with cell phones. Training of supervisors on the use of the management console was more challenging for some as it involved a higher level of computer literacy. All three organisations reported that their biggest challenge was ensuring appropriate use and proper care of the mobile hand set – which required new human resource policy and strict enforcement. One manager reported that:

"“People were losing their phones or it was stolen … allowing their children to play with them, not taking care, like getting it wet. Initially it was hard to find the right balance to be fair and make sure they take responsibility for the phone”. (Interview, CMT programme manager)"

#### Technological systems

The organisations reported very few problems related to the new technology. They reported that CHWs found the technology acceptable and useful for their work. Digital providers emphasised the importance of ‘use-ability’ for sustaining effective use of the mobile management system. One digital provider who designed the system for the CMT organisation noted:

"“If CHWs do not see a direct benefit for their work, such as reduction in the time it takes to document things or getting useful feedback from supervisors, the technology will not be used effectively”. (Interview, digital provider)"

Entry level phones were sufficient for the required tasks. CHWs were familiar with this level of mobile phone technology and it also kept the cost of purchasing mobile handsets low. The mobile software applications and support services were designed by local digital providers (non-profit and for-profit), attesting to the highly developed mobile phone industry in South Africa. Both open-source and proprietary software systems were used. There were no major requirements for interoperability of the mobile phone system with existing information systems (due to the small scale of the projects). The organisations were able to protect the security of their data with access control codes that allowed for different levels of access for CHWs and managers.

One NGO reported experiencing technical problems due to unreliable internet access. This NGO also advised that more time should be allocated for mHealth implementation projects as it took longer than planned and required adjustments along the way. They also noted that not being able to capture qualitative data was a limitation and that indigenous language options should be made available [29].

#### Financial systems

The mHealth interventions were initiated with the help of donor funding, which may pose a challenge for sustainability in the long term. Information on costs was limited and where available it was difficult to interpret as some of the technology was subsidized by the digital providers and none of the projects evaluated the cost-effectiveness. The one evaluation study that was done focused on feasibility and efficiency gains only and not on impact on effectiveness of core activities [29,30].

To summarise, the key informant interviews and local case studies established the facts of how mHealth is implemented and the local benefits and challenges of current mHealth interventions for CBS in South Africa. The organisations and digital providers in these examples represent a small number of role-players who, with the support of donor funding, are learning rapidly and circulating knowledge on their experiences. The case examples described in Table 4 illustrate the benefits of mHealth applications for M&E and for supporting and developing management and supervision of CBS in a research and service delivery setting. The few challenges identified related to ensuring responsible use of the mobile phones and in one case, unreliable internet connectivity.

Whilst the positive experience of these organisations attest to the enormous potential for mHealth to strengthen health systems in South Africa, what remains unclear, is the extent to which this new technology can achieve a fit with the social, technological and organisational dimensions of the current public sector health system. As is the case in the broader literature, the examples reviewed here, are small scale, donor funded, sometimes short-term and not integrated into the mainstream health system [2,4,18]. What we do not know is whether benefits witnessed in these local case examples (and those in the broader literature) would be retained and what might be the opportunities and challenges of implementation at scale in a routine public sector environment.

### Appraising health systems challenges for mHealth for CBS in South Africa

#### Stewardship

The lack of high level strategic, policy and financial support from governments is a key reason for the absence of large scale government sponsored mHealth projects [3,4,6,11,19,21,25,34]. In South Africa, the national health ministry is increasingly taking a stewardship role as part of a renewed focus on improving health information systems. The emergence of new technological platforms such as a web-based routine district health information system (DHIS), an electronic record system for antiretroviral treatment and an eHealth strategy, are examples of leadership in the health information field. The first question - is there a policy environment supportive for mHealth, appears largely satisfied in the South African context.

Nevertheless, there are other stewardship challenges that include alignment of policy with and integration into health sector plans, strategies and systems [3,4]. This includes leadership and coordination in finding workable solutions to interoperability, (through for instance, developing common standards and national regulations), as well as identifying workable and affordable open-source and or proprietary software solutions. Stewardship tasks that will require long-term commitment include developing an evidence base to document and learn from best practices, identifying sustainable funding sources and creating partnerships with NGOs and the private sector to assist with future implementation [2-4,6,17,18,21].

#### Organisational systems

There are a range of system and structural issues at other levels that could challenge the successful implementation of mHealth solutions at scale [2-4,6,11,17,18,20,21]. A recent (2011) report evaluating the implementation of national eHealth strategies in Europe noted that “the complexity of eHealth as a management challenge has been vastly underestimated” [35].

The South African health system has been characterised as weak compared to other LMIC countries that spend a smaller proportion of their gross domestic product (GDP) on health care and show better health outcomes [36]. Whilst part of the comparatively poor health outcomes are ascribed to the high HIV &TB burden of disease (and to some extent also due to the existence of two systems, public and private), health system weaknesses contributing to poor outcomes include poor governance, management and accountability systems, and a still weak PHC system with inadequate access and quality of health care. The result of these organisational weaknesses is a gap between new policy formulation and effective implementation, including for introduction and use of new information technology [10,21,36,37].

One organisational challenge is how to effectively align the use of mobile phone technology with the strategic goals and priority interventions of national and provincial health departments [3,4,6,21]. In South Africa, a major area of health system weakness is the poor capacity of provinces and districts to use health information for management [5,10,36] and this could limit the potential value of new mobile technology. In a large, multi-country WHO/Health Metric Network assessment in 2009, South Africa scored below 50% in key areas such as overall availability of health information resources, data management and use of information for implementation and action [38]. These gaps were confirmed in a recent (2011) rapid needs assessment of the routine district health information system that showed a low capacity for, and little time spent on, health information for management [39]. The NDOH report also highlighted an environment where ICT was weak and not firmly entrenched – with inadequate availability and use of ICT (such as computers, the internet, updated software) and deficiencies in the management and maintenance of these services. Further, while there are a few examples of provincial electronic health records systems, the country is far from establishing a national patient health record system [5].

Supervision of health workers in South Africa and other low- and middle-income settings have been characterised as weak [10,40]. Fragmentation associated with delivery of community-based services by NGOs could be considered as adding a further structural weakness in the supervision of community-based services. The range of problems with CBS supervision in South Africa include a lack of adequate and standardized health information tools and processes, lack of integration of information into the existing routine health information system, lack of dedicated M&E staff and budgets and inadequate training at all levels [8,29,41-43].

With the planned PHC re-engineering, the supervision of CBS will shift from an NGO base to local public sector players. This is of concern as the current PHC facility supervision systems are themselves weak and in need of intervention. For instance, in 2010, some districts had less than half the required PHC supervision visits per year [10]. The broader reasons for inadequate supervision of health personnel in LMIC settings include a range of obstacles at multiple levels of the health system such as low management skills, poor co-ordination, lack of motivation, inexperienced and untrained staff, obstacles that will require broader health systems changes [40,44,45].

MHealth experts have cautioned against regarding mobile technology as an intervention that will solve problems of poorly functioning systems [4] and it may be unrealistic to expect a mobile management system to improve supervision problems in the context of a struggling PHC system in South Africa. In addition, the quasi-federal provincial governance system makes for a complex implementation environment where provinces are not compelled to follow national government improvement plans and where uncertain funding streams could further threaten the sustainability of large scale mainstreamed mHealth projects.

#### Technological systems

Ensuring the use-ability of mobile phone management systems may present a challenge for a large-scale mHealth for CBS project, given the variety of stakeholders involved [4,11,21]. Whilst use-ability of front-end users (the CHWs) have been highlighted, perhaps as big a challenge is ensuring that supervisors and managers at local, district, provincial and national levels experience the mobile phone technology (and the information generated) as useful. This would require extensive training on the use of the management systems as well as building technical capacity for maintaining and fixing mobile systems [4,11,19,21].

An interoperable mHealth system needs to ensure that various routine and other information systems are able to connect, ‘communicate’ and share information [3,18,21,25]. Examples include transmitting data collected in community-based services to the routine health information system, the linking of community and clinic–based electronic patient records in a way that allows for tracking bi-directional referral, follow-up care and prescribing of medicine. The technological and organisational challenges to satisfy the interoperability and privacy requirements [1,3,4,21] of a newly integrated large-scale CBS would be considerable, especially given the multiple levels of access required for various levels of health workers.

#### Financial systems

As mentioned earlier, securing sustainable funding for large-scale implementation is one of the stewardship functions of government, one that could represent a major challenge [3,4,11,19]. Plans for implementation and sustainability of mHealth projects would need to be realistic, especially as ICT projects may cost more and take longer than initially planned [3,25]. One of the digital providers interviewed spoke to the broader challenge of financial planning when he referred to the importance of achieving the ‘Goldilocks zone’ – a place where the present requirements of the technology, the scope for future expansion of the technology and its affordability, are perfectly matched with the requirements of the client seeking to implement mHealth. Achieving the ‘Goldilocks zone’ for a large-scale mHealth intervention for M&E of CBS will no doubt be harder than for smaller organisations.

In the absence of evidence on cost–effectiveness of mHealth strategies at scale [4], it is also difficult weigh-up mHealth interventions against other priority and evidence-based interventions in South Africa (in terms of the costs, resources and capacity requirements). There may be unintended negative consequences of introducing new technology within a weak health system [4], such as staff avoiding engagement with health information systems that are not functioning properly. These and other implementation challenges would have to be monitored alongside evaluation of cost-effectiveness. However, such evaluations have been absent from other ICT interventions in South Africa. For example, the majority of telemedicine projects implemented in the past decade failed to survive past the pilot phase, prompting the South African NDOH in 2010 to put a moratorium on the implementation of new telemedicine projects until a strategy could be found to increase the success rate [22]. This illustrates the complexity of implementing and sustaining modern ICT in this setting.

## Conclusion and recommendations

Against a background of increasing enthusiasm for the use of mobile phone technology in health services in LMICs, this paper reviewed the benefits of mHealth for the monitoring and evaluation of CBS and the challenges of scaling this up for mainstream health services in South Africa. We identified the need for a health systems perspective that could guide our appraisal of the challenges, one that would address the range of health systems complexities, whilst also integrating the technological issues that need to be addressed. The paper proposed a health systems framework that focused attention on the broader health systems dimensions of stewardship, organisational, technological and financial systems when considering mHealth at scale for community based health care - a framework that is generic enough to apply to other mHealth and eHealth applications, as well as to other LMIC settings.

In sum, our analysis suggests that although mobile phone management systems can benefit the monitoring and management of service delivery in CBS, it is uncertain that these benefits would be realized and or sustained for large-scale mainstreamed mHealth for CBS programmes. South Africa has a positive environment for mHealth implementation that includes a high prevalence of mobile phones, a well developed ICT industry, examples of small-scale successful use of mHealth for CBS and a government supportive of eHealth development. Nevertheless there are major weaknesses in the functioning in the public sector PHC system that could jeopardize the successful implementation and value of mHealth programs. Challenges to scaling up exist in all four of the health systems dimensions (stewardship, organisational, technological and financial). Chief amongst these are the weaknesses in organisational capacity and culture of using health information for management and a still weak ICT environment.

Against the background of a struggling health system with uncertain implementation capacity and the lack of an evidence base on cost-effectiveness of large scale mHealth solutions, it would seem wise to not opt at this stage for full-scale use of mobile management systems for M&E of CBS in South Africa. Rather, we recommend that South Africa adopt a developmental approach to the implementation of mHealth. In selected areas where organisational capacity for implementation exist, the health department could follow a building blocks approach that involves encouraging the initial implementation of smaller, phased and heavily evaluated ‘lead’ projects, within the routine service environment. Implementation should pay particular attention to the technological issues of end-user acceptability, interoperability with both technical and human resource systems as well as ensuring security and privacy of patient information. This will allow for growing the capacity for implementation and the evidence base on mHealth in mainstream health settings - evidence that can in turn inform future developments in policy and practice.

A limitation of this study is that evaluating challenges of scaling up was complicated by the lack of an evidence base on effectiveness of mHealth and absence of guidance on effective implementation of new ICT in health services. The interviews and case examples of organisations using mHealth showed a positive appraisal of mHealth and this view does not take account of those who may have had a less positive experience, nor does it take account of mobile applications for purposes other than monitoring and evaluation of CBS. In the absence of a systematic evidence base, the authors therefore had to rely on a range of methods and a theoretical consideration of health systems challenges, to generate knowledge that could be used to guide decision-making on mHealth policy and practice in South Africa.

This review was limited to using mobile technology systems for the M&E for CBS. Whilst there is stronger evidence of effectiveness of using mHealth interventions for delivering preventive health messages and promoting adherence to health care and medication, it is likely that scaling up of such interventions in LMICs will be faced with similar implementation challenges.

To conclude, the message from this investigation is that, in addition to the need for more evidence on effectiveness, policy makers and implementers would benefit from considering the broader health systems dimensions of up-scaling mHealth. The proposed health systems framework can assist with such deliberations. It also can assist with demystifying the complexities and the ‘hype’ associated with the field of mHealth - by refocusing it as a health systems issue rather than an exclusively technical question of information technology. Recommendations flowing from applying such a framework will be able to more holistically address the appropriateness and ‘fit’ of mobile phone technology within a health system, and how such technology may be employed to add value in improving health systems and health outcomes.

## Competing interests

The authors declare that they have no competing interests.

## Authors’ contributions

HS, ED, NL conceived of the study and participated in its design and coordination. NL was responsible for the acquisition, analysis and interpretation of data. NL, HS and ED have been involved in drafting the manuscript and revising it critically for important intellectual content. All authors read and approved the final manuscript.

## Pre-publication history

The pre-publication history for this paper can be accessed here:

http://www.biomedcentral.com/1472-6947/12/123/prepub
